# Compliance with protective lung ventilation in an Irish teaching hospital

**DOI:** 10.1186/cc13485

**Published:** 2014-03-17

**Authors:** S Chong, E Moloney, M Donnelly, G Fitzpatrick

**Affiliations:** 1 Tallaght Hospital, Dublin, Ireland

## Introduction

The importance of protective lung ventilation in reducing mortality in adult respiratory distress syndrome (ARDS) patients is well described [1]. However, suboptimal compliance with the recommended tidal volumes has been reported [2]. Therefore, we wished to assess our compliance in adhering to protective lung ventilation in patients with, or at risk of developing, ARDS.

## Methods

A retrospective review was done on all mechanically ventilated patients in the ICU of Tallaght Hospital over a 6-month period (February to July 2013). Hourly tidal volumes were recorded automatically in electronic charts. Compliance was assessed by calculating the total time patients with, or at risk of developing, ARDS were ventilated with tidal volumes < 6 ml/kg, 6 to 8 ml/kg, and >8 ml/ kg during the first 72 hours on mechanical ventilation. ARDS is defined as per the Berlin criteria [3]. Exclusion criteria were patients who did not receive invasive ventilation or who were ventilated for less than 72 hours. We also assessed whether patients' height was documented.

## Results

A total of 72 patients were ventilated for >72 hours. Of these patients (44 males, 28 females, mean age 65.5 years), ARDS criteria were met in 17 patients and 22 patients were determined to be at risk of developing ARDS. For patients with ARDS, the ventilated time with tidal volumes <6 ml/kg, 6 to 8 ml/kg, and >8 ml/kg was 25.3%, 31.7% and 43% respectively. For patients at risk of developing ARDS, the ventilated time with tidal volumes <6 ml/kg, 6 to 8 ml/kg, and >8 ml/ kg was 15.7%, 29.3%, 55%, respectively (Table 1). A total of 16 patients (nine who had ARDS) had no documentation of their height.

**Table 1:** 

Tidal volume	With ARDS (%)	At risk of ARDS (%)
< 6 ml/kg	25.3	15.7
6 to 8 ml/kg	31.7	29.3
> 8 ml/kg	43	55

## Conclusion

Compliance with protective lung ventilation in our ICU is suboptimal. This may be due to the lack of education and guidelines in the unit regarding protective lung ventilation. Moreover, accurate recording of patient height and determination of predicted body weight should be documented to facilitate accurate tidal volume calculation and protective lung ventilation.
